# Evaluation of a Digitally Guided Self-Rehabilitation Device Coupled With Telerehabilitation Monitoring in Patients With Parkinson Disease (TELEP@RK): Open, Prospective Observational Study

**DOI:** 10.2196/24946

**Published:** 2022-02-07

**Authors:** Margaux Blanc, Anne-Laure Roy, Bastien Fraudet, Patrice Piette, Elodie Le Toullec, Benoit Nicolas, Philippe Gallien, Emilie Leblong

**Affiliations:** 1 University Hospital Center (CHU) Rennes University of Rennes Rennes France; 2 Hospital Center (CH) Saint Malo Saint Malo France; 3 Physical and Rehabilitation Medicine (MPR) Pole Saint Helier Rennes France

**Keywords:** Parkinson's disease, telerehabilitation, serious games, UTAUT, physiotherapist, acceptability, acceptance

## Abstract

**Background:**

Parkinson disease is a neurodegenerative disease causing a progressive loss of autonomy. This requires long-term rehabilitation care. Currently, new technologies are being developed for use in daily life, and there is a progressive implementation of telerehabilitation.

**Objective:**

The aim of this study (the TELEP@RK study) is to evaluate the uses of a digital self-rehabilitation device in patients with Parkinson disease and their independent physiotherapists on the scale of a health territory.

**Methods:**

A total of 10 independent physiotherapists and 31 patients with Parkinson disease were followed for 1 year to evaluate the use of a telerehabilitation tool (digital tablet and inertial sensor) via questionnaires of the Unified Theory of Acceptance and Use of Technology (UTAUT). The questionnaires were submitted to participants at 0, 2, and 12 months from the start of follow-up. The averages of the scores of the different determinants and constructs of the UTAUT questionnaires were compared at the different follow-up times.

**Results:**

Among professionals, the averages of the various determinants were generally high at the beginning of the study with an average (out of 5) performance expectancy of 4.19, effort expectancy of 3.88, social influence of 3.95, facilitating conditions of 4, and intention to use of 3.97. These averages decreased over time.

**Conclusions:**

Acceptability, acceptance, and appropriation of the tool were very high among the physiotherapists as well as the patients, despite the tool’s lack of evolution during the study. In the current health care context, these results allow us to envision a new organization of the care pathway for patients with chronic diseases, with the increased use of new technologies associated with telecare.

## Introduction

Parkinson disease is a neurodegenerative disease characterized by progressive motor and nonmotor symptoms. There is no curative treatment, but there are symptomatic treatments, including medication (dopaminergic treatment) and surgery (deep brain stimulation). Even with optimal treatment, there is a progressive deterioration of autonomy (sensitive non-dopa symptoms: posture, walking, and balance) which affects the patient’s quality of life [1].

Rehabilitation plays an important role in the management of Parkinson disease and is considered complementary to drug and surgical treatments. This is why it is important to offer patients rehabilitative care as soon as possible [2,3]. It has been shown that exercise (eg, aerobic exercise, stretching, strengthening exercises, balance exercises, dance, resistance training, and walking) has positive effects on the quality of life of patients [4] and improves motor and nonmotor symptoms [5,6]. In addition, a review of the literature [7] suggests that physical activity improves the ability to perform activities of daily living, especially in the early stages of Parkinson disease. Self-rehabilitation, which consists of the autonomous practice of exercises at home, is becoming increasingly common, most often as a relay to hospital care or as a complement to outpatient care, and appears to be at least as effective as center-based rehabilitation [8]. Among patients with Parkinson disease, those who benefit from ambulatory rehabilitative care seem to have an improved quality of life [9].

At the same time, due to the development of new technologies for use in daily life, several studies [10,11] have evaluated tools aimed at offering independent home rehabilitation via rehabilitation games. The term *serious games* refers to game software combining serious aspects (eg, software with educational, informational, communicational, marketing, ideological, or training purposes) with interactive and playful aspects of video games [12]. The use of such tools seems to be effective on motor symptoms [13-15]. Several serious game programs have been tested, and their use by patients with Parkinson disease seems feasible [14,16-18]. The Beyond Your Motion (BYM) tool is a tool coupling a serious game via an app on a digital tablet to a motion sensor.

We are also witnessing the development of telerehabilitation, which is defined as a service using telecommunications technologies to provide remote rehabilitation services. A review of the literature [19] highlights an improvement in functional activities, symptoms (pain, insomnia), and quality of life in multiple sclerosis patients following telerehabilitation. Studies have shown its feasibility and safe application for stroke patients [20]. Since the beginning of 2020, the COVID-19 pandemic has led to increased use of the digital tools offered by telemedicine to ensure the safe continuity of care. In the context of this health emergency, new decrees have been issued to facilitate the use of these tools, notably with reimbursement, in the same way as face-to-face procedures, through health insurance for telecare and teleconsultations [21].

**Figure 1:**
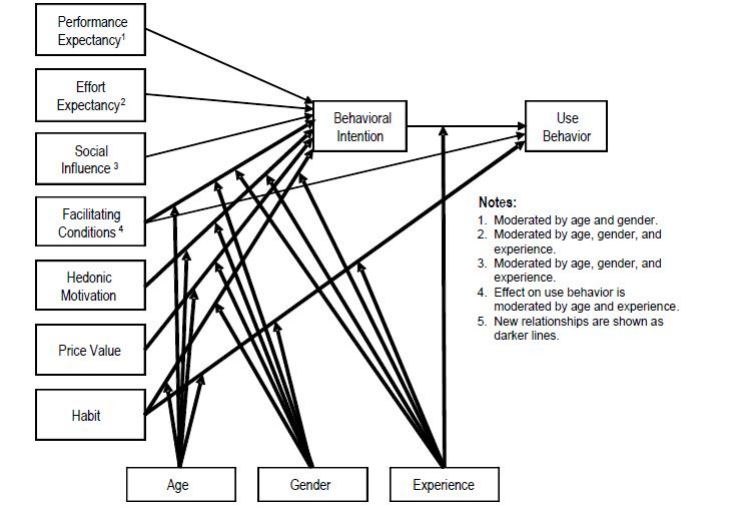
Unified Theory of Acceptance and Use of Technology 2 (UTAUT 2) model. Image taken from Venkatesh et al [25].

The use of new technologies is at the center of daily life. This use is intimately linked to the acceptance of these tools. The purpose of studying use is to evaluate how people appropriate and use products over time through three different processes: acceptability, acceptance, and appropriation. Acceptability corresponds to the subjective representation of the use of the technological tool before it is used. Acceptance refers to the experience (judgment and behavioral reactions) of individuals after the introduction and use of the product. Appropriation thus corresponds to the process by which the person invests in the product and the extent to which the product is in line with their personal and cultural values that make them want to act on or with the product and not just be subjected to its use [22]. Thus, we can speak of a continuum between these different notions, starting before use and continuing to prolonged use [23]. This process of acceptability has been the subject of numerous theories, some of which have led to explanatory theoretical models. The Unified Theory of Acceptance and Use of Technology (UTAUT) [24] is an integrative model (a review of 8 theoretical models) that synthesizes these different approaches. A total of 7 constructs were considered to be significant determinants of intent to use, and these were grouped into 4 determinants: (1) performance expectancy (the degree to which an individual believes that using the product will help them achieve a goal), (2) effort expectancy (the degree of ease associated with using the product), (3) facilitating conditions (the degree to which an individual perceives that an organization and support exists to help them use the product), and (4) social influences (the degree to which an individual perceives that people important to them believe that they should use the product). In addition to these constructs are moderators such as age, gender, experience, and voluntarism. These different constructs act on behavioral intention, under the influence of moderating variables, which leads to use behavior. The results of this model showed an explanatory power of the intention to use behavior of about 70%. A new version of the UTAUT, UTAUT 2, was also proposed in 2012 [25] and has 3 additional constructs, namely hedonic motivation, price value, and habits (Figure 1) [25].

The aim of this study (the TELEP@RK study) is to evaluate the use of two rapidly developing approaches (serious games and telerehabilitation) by patients with Parkinson disease and their physiotherapists.

## Methods

### Scheme of the Study

**Figure 2:**
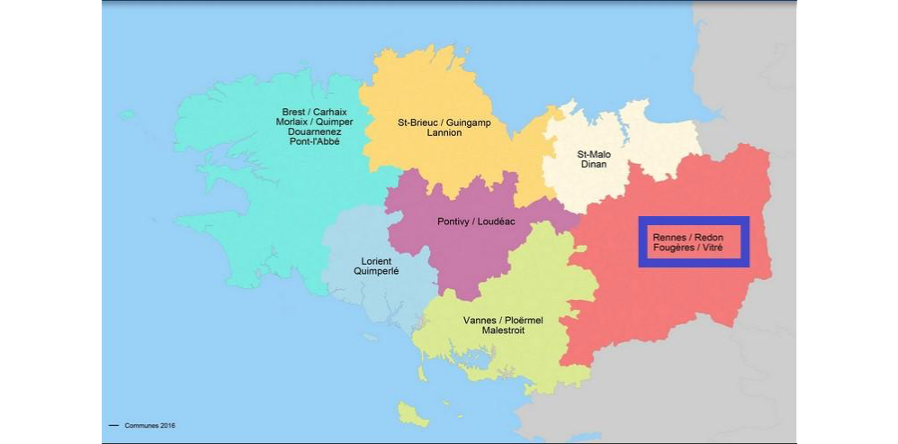
Health territories of Brittany, France. Territory number 5 is enclosed in the blue rectangle. Image taken from Agence Régionale de Santé Bretagne [26].

This is an open, prospective observational study that took place from 2017 to 2020 over health territory 5 in Brittany, France (Figure 2) [26]. The patients were followed for 12 months.

The main evaluation criterion was the questionnaire on the acceptance of the tool and its use by professionals based on the 2-month UTAUT.

The secondary judgment criteria were:

- Professional and patient (and patients’ relatives) acceptability questionnaires at baseline.
- Questionnaire on acceptance of the tool by patients based on the UTAUT at 2 months.
- Rosenberg self-esteem score, which is a factor in social integration, at baseline, 2 months, and 12 months.

Pôle Saint Hélier, a physical medicine and rehabilitation center in Rennes, sponsored this study.

### Population Studied

The criteria for inclusion differed based on the type of subject. For health professionals, the inclusion criteria were as follows: physiotherapists, physical and rehabilitation medicine hospital physicians, or independent practitioners in health territory 5 in Brittany, France, who agreed to participate in the study. For patients, the sample included men and women over 18 years of age presenting idiopathic Parkinson disease or a related syndrome, presenting a complaint concerning balance and/or walking on a perimeter of at least 100 meters with or without technical aid, at stage 4 or less on the Hoehn and Yahr scale, and formulating their free and informed consent in writing. The patients had not received prior rehabilitation in a rehabilitation center. The sample excluded patients with orthopedic and rheumatological pathologies that might prevent the performing of the measurements or comprehension disorders that might prevent the performing of the protocol. The first inclusions began in May 2018, and the study ended in June 2020.

### Digital Tool

**Figure 3:**
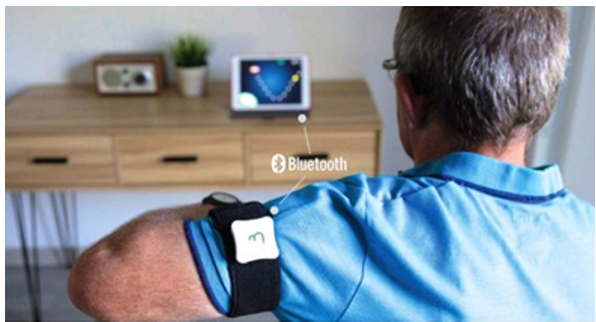
The Beyond Your Motion (BYM) tool. Image taken from the BYM website. [27]

An interactive digital tool (tablet and inertial sensor; Figure 3) [27] selected by physicians or physiotherapists to support the self-rehabilitation of patients with Parkinson disease at home was made available to patients in this study. This tool was a digital support comprising bracelets equipped with motion sensors and a tablet with a serious game app with visual and auditory feedback for self-rehabilitation and reconditioning to autonomous effort. This was a tablet app that guided the patient’s active work. In particular, the parameterization allowed the app to work according to predefined levels of difficulty, but in a self-learning form: depending on the patient’s progress, the difficulty increased according to several parameters (duration of the session, frequency of the exercise, size of the pointer, etc). It was coupled to a miniaturized movement analysis device using inertial control units that the patient could position alone using Velcro strips, as indicated by the exercises, on either side of a joint. The patient performed repetitive movements with the largest possible amplitude with a defined pace and received visual feedback through a visual of a vessel catching coins and stars, and auditory feedback, according to the movements performed. The exercises were designed to target motor symptoms such as bradykinesia and hypokinesia. The connection with the field professional was made via the secure platform used for telemedicine. It was anticipated that the monitoring and regulation of self-rehabilitation would be done remotely by telerehabilitation (part of the regulatory framework of telemedicine).

### Conduct of the Study

Physiotherapists were contacted by mail or email and invited to an information session at which they could give their consent to participate in the study. The BYM tool was made available free of charge for 1 year to the physiotherapists who had given their consent. The physiotherapists recruited patients with Parkinson disease from among their patients. Patients were offered a medical consultation by a Pôle Saint Hélier investigating physician to verify the inclusion and exclusion criteria and they were included in the study after obtaining their informed consent. Physiotherapists had the choice of using the tablet in their office or in the patient’s home.

The evaluation was done in the form of questionnaires. The study subjects, as well as their relatives and the professionals taking care of them, were required to fill out these questionnaires at baseline, 2 months, and 12 months from the beginning of the study.

### UTAUT Questionnaires

The distribution of the various items and constructs was modified after data collection for the statistical analysis. It should be noted that the item concerning the price was not asked of the independent physiotherapists at the beginning of the follow-up.

### Statistical Analyses

We analyzed the data using the statistical software R (R Foundation for Statistical Computing). A first descriptive analysis was performed with numbers and percentages for the qualitative variables and means, standard deviations, ranges, and medians for the quantitative variables. The Wilcoxon test was used to compare the various means. The main criterion of judgment, obtained via use evaluation questionnaires (UTAUT), is quantitative. The analysis of this criterion consists of the comparison of the means obtained at baseline and at 2 months using the Wilcoxon test. All tests were performed with a significance threshold of 5% (*P*<.05). We applied imputation models for missing data when necessary.

Patients gave their free, informed, and written consent to participate in the study. The study protocol and an information and nonopposition note to the study were submitted and accepted by the CPP Sud Est II Lyon Bron ethics committee on December 13, 2017 (2017-A02834-49, RIPH 2).

## Results

### Study Population

A total of 72 independent physiotherapists were contacted based on the directory of professionals who have taken training courses at the Réseau Park through the NeuroBretagne Association. Of the contacted physiotherapists, 13 responded to the invitation to participate in the study and were preincluded. Then, 380 independent physiotherapists were contacted via the Union Régionale des Professionnels de Santé. Of the 380, 2 responded. Among the preincluded physiotherapists, 5 were excluded because they did not recruit any of their patients for the study. Of the 10 physiotherapists included, 3 (30%) were men and 7 (70%) were women. The average age was 39.3 years (SD 10.2 years). The average number of years of experience as an independent practitioner was 12.7 (SD 10.7).

A total of 31 patients were included in the study by their independent physiotherapists, including 17 men (55%) and 14 women (45%). The mean age was 69 years (SD 8 years). Of the 31 patients, 28 had idiopathic Parkinson disease, 2 had progressive supranuclear paralysis, and 1 had an extrapyramidal syndrome of undetermined etiology. The average duration of disease progression was 7 years (SD 5 years). The average stage on the Hoehn and Yahr scale was 1.87 (SD 0.86), indicating moderate disease impact in these individuals, all living at home. No participants had deep brain stimulation electrodes implanted. The mean motor Unified Parkinson’s Disease Rating Scale (UPDRS) score was 10.9 (SD 6.97) and the score for Motor Aspects of Experiences of Daily Living (UPDRS M-EDL scale) was 10.4 (SD 7.3). Out of the 31 patients, a total of 17 (55%) already had a tablet, 16 (52%) had a smartphone, and 27 (87%) had internet access. Among those with a smartphone and/or tablet, 17 of 19 (89%) used their devices regularly or daily. A total of 4 (13%) of the 31 patients lived alone, 27 (87%) lived with a family caregiver, and 2 (6%) had professional caregivers. For 27 of the 31 patients (87%), shifting was considered easy with or without aids. Out of the 31 patients, 12 patients (39%) said they lived far from a physiotherapy practice and 7 (23%) were far from a rehabilitation center. See Table 1 and Table 2 for patients’ baseline demographic and clinical characteristics.

Initially, it was anticipated that self-rehabilitation would be monitored and regulated remotely at the request of the independent physiotherapist. However, there were no requests for teleconsultation by the independent physiotherapists during the study.

**Table 1:** Patients’ baseline demographic and clinical characteristics (n=31).

Characteristics	Mean (SD)	Range
Age, years	69 (8)	50-83
Hoehn and Yahr stage	1.87 (0.86)	1-4
Duration of disease progression, years	7 (5)	1-20
Unified Parkinson’s Disease Rating Scale: Motor	10.9 (6.97)	1-24
Unified Parkinson’s Disease Rating Scale: Motor Aspects of Experiences of Daily Living	10.4 (7.3)	1-36

**Table 2:** Patients’ baseline demographic and clinical characteristics, continued (n=31).

Characteristics			Patients, n (%)
**Sex**			
	Male	17 (55)	
	Female	14 (45)	
Numeric tablet			17 (55)
Smartphone			16 (52)
Internet			27 (87)
**Disease**			
	Parkinson Disease	28 (90)	
	Progressive supranuclear paralysis	2 (7)	
	Undetermined	1 (3)	
**Use of tablet and smartphone**			
	Never	0 (0)	
	Sometimes	2 (10)	
	Regularly	10 (53)	
	Daily	7 (37)	
**Daily living context**			
	Alone	4 (13%)	
	Family caregiver	27 (87)	
	Professional caregiver	2 (6)	
**Shifting difficulty**			
	Easy without help	22 (71)	
	Easy with help	5 (16)	
	Difficult without help	2 (6)	
	Difficult with help	2 (6)	
Home far from physiotherapist’s practice			12 (39)
Home far from rehabilitation center			7 (23)

### Results for the Main Criterion

Concerning the evaluation of use via the UTAUT questionnaire among professionals, the means of the various determinants were globally high at the beginning of the study, with average scores (out of 5) as follows: performance expectancy of 4.19 (SD 0.33), effort expectancy of 3.88 (SD 0.57), social influence of 3.95 (SD 0.60), facilitating conditions of 4 (SD 0.62), and intention of use of 3.97 (SD 0.66).

There was a decline in the averages of the different determinants and constructs over time.

There was a significant decline (*P*=.03) in the means of perceived usefulness between the beginning and end of follow-up. The mean at month 0 was 4.25 (SD 0.38), while the mean at month 12 was 3.76 (SD 0.45). This decline predominated between the 2nd month and the 12th month (*P*=.049); the mean at month 2 was 4.1 (SD 0.34). This particularly concerned the usefulness of the tool in the office (*P*=.04) and in the patient’s home (*P*=.04) between the 2nd and 12th months of tool use. The job fit, as in, the use in daily practice, decreased significantly between the beginning of follow-up and 12 months of use (*P*=.001), with a more marked decrease between 0 and 2 months of use (*P*=.02) (mean at month 0 was 4.1, SD 0.45; mean at month 2 was 3.56, SD 0.47; mean at month 12 was 3.04, SD 0.71).

Intention to use also decreased over time. The mean at month 2 was 2.84 (SD 0.88), while the mean at month 12 was 2.14 (SD 0.63); there was a significant difference between month 0 and month 2 (*P*=.007), and between month 0 and month 12 (*P*<.001).

For the social influence determinant, physiotherapists believed that patients became increasingly less favorable toward the use of this tool, with a significant difference (*P*=.04) in the means between the 2nd and 12th month of use. The mean at month 2 was 3.92 (SD 0.6), while the mean at month 12 was 3.47 (SD 0.57).

The means of the credibility construct decreased between the beginning and end of follow-up (*P*=.005), especially between 2 and 12 months of use (*P*=.01). The mean was 3.9 (SD 0.88) at month 0, 3.8 (SD 0.52) at month 2, and 3.23 (SD 0.55) at month 12.

The averages of the effort expectancy and price value determinants were stable. The average effort expectancy at month 0 was 4 (SD 0.61) and that at month 12 was 3.71 (SD 0.25), for *P*=.26. The average price value at month 2 was 2.53 (SD 0.96) and that at month 12 was 2.32 (0.87), for *P>*.99.

### Results for the Secondary Criteria

Concerning the evaluation of the determinants and constructs of UTAUT in patients, the averages were also high initially, with an average (out of 5) performance expectancy of 4.14 (SD 0.55), effort expectancy of 4.22 (SD 0.69), social influence of 4.21 (SD 0.66), facilitating conditions of 4.18 (SD 0.59), price of 2.84 (SD 0.97), and intention of use of 3.53 (SD 0.96).

There was a decrease in the averages of the various constructs and determinants over time.

The averages for the performance expectancy determinant decreased throughout the study (*P*=.004), especially between the beginning of the study and 2 months of use (*P*=.02). The average at month 2 was 3.81 (SD 0.64), and that at month 12 was 3.75 (SD 0.55). It could be observed that patients find the tool less and less useful; the mean of the perceived usefulness construct was 4.09 (SD 0.60) at month 0 and 3.71 (SD 0.56) at month 12. The reduction in the perceived usefulness of the tool was especially true for intense exercise (*P*<.001), for patients to see themselves progressing (*P*=.01), and for use at home (*P*=.002), between the beginning of the study and 12 months. On the other hand, the perception of the usefulness of auditory feedback was better (*P*=.002) at 2 months, with a mean of 4.16 (SD 0.82), compared to the beginning of the study, when the mean was 3.58 (SD 1.03).

The intention to use decreased (*P*=.02) over time, especially between the 2nd month, when the mean was 2.54 (SD 1.08), and the 12th month of use, when the mean was 1.95 (SD 0.73).

Moreover, various determinants and constructs were stable during the study.

Initially, patients’ self-esteem self-assessment was low, with a mean of 25.5; at the end of the study, it was considered very low, with a mean of 24.3 (Table 3).

**Table 3:** The change in the patients’ self-esteem evaluated using the Rosenberg Self-Esteem Scale.

Month of follow-up	Mean self-esteem value (SD)	Range
Month 0	25.5 (2.6)	19-31
Month 2	26.3 (2.8)	21-34
Month 12	24.3 (2.6)	19-30

## Discussion

The scores obtained by the UTAUT questionnaires were globally very high (higher than 4 out of 5 for most of the determinants), which is synonymous with good acceptability, acceptance, and appropriation of the tool. Over time, there was a downward trend in the various scores, which can be explained by many factors.

### Important Expectations of the Tool

The fact that the performance expectancy is initially high is synonymous with high expectations on the part of the physiotherapists. This must be placed in the context of a neurodegenerative disease where physiotherapists can sometimes feel powerless against the inevitable degeneration of their patients’ conditions. Over time, they may have been disappointed, which was probably related to a weariness of the app interface that did not evolve during the study (the serious games that had been developed for the prototype remained the same during the year the participants were followed). This phenomenon can be described as a wow effect. In marketing, this phenomenon corresponds to an initial craze for new technologies and is very frequent and well described [28].

The decrease in patients’ intention to use the tool can be explained by the fact that the wow effect is more pronounced in chronically ill patients because they are typically waiting for a treatment that will stop the progression of their disease. Regular updates, different game interfaces, and web-based challenges between different patients could improve long-term adherence.

The decrease in self-esteem is statistically significant, but this is not clinically relevant because it only decreases by 1 point on average. There is only one low extreme value that does not explain alone that the average goes from low (between 25 and 30) to very low (<25). In addition, Parkinson disease is a neurodegenerative disease whose natural progression cannot be stopped by the use of a rehabilitation tool.

### A Tool That is Easy to Use for Parkinson Disease and Remains So

The effort expectancy was stable during the study, which demonstrates that the activities proposed by the tool are easy and accessible to this population of patients with Parkinson disease.

The fact that the averages of the social influences determinant and the image construct decrease over time is rather positive. Once the wow-effect is over, appropriation does take place because patients consider the tool part of their daily life and unexceptional.

The scores concerning the usefulness of visual and auditory feedback (increasing significantly between month 0 and month 2) are very good, which confirms the importance of using feedback controls for the rehabilitation of gait and limb motor disorders in patients with Parkinson disease [29].

The exercises are well explained, and the activities are easy to understand (stable and very high scores), making the tool easy to use. It reflects a positive image of rehabilitation and physical activity. It is a playful and adaptive tool with an adapted progressiveness (scores of the facilitating conditions are very high and stable).

Patients experience greater benefits when using the BYM tool compared to paper exercises and exercises alone. (very high and stable scores for the relative advantage construct).

### A Study Impacted by the Economic Context of BYM

The BYM company experienced financial difficulties during this study, which resulted in a hindrance to the development and evolution of the product. This explains the decrease in the playful aspect (weariness of the proposed exercises) and credibility (lack of progressiveness) constructs.

The scores concerning the price are biased because the devices were free of charge during the study.

The decrease in intention to use can be explained by the lack of evolution of the tool and the absence of after-sales service. The study took place over 2 years (1 year of recruitment and 1 year of follow-up), which is an important length of time considering the economic context of start-ups. These results raise the question of the adaptability of clinical research methods in France to economic models that are increasingly fast and flexible to bring innovation to the health care sector.

### A Recent Change of the Health Context

The last questionnaires for patients and their private physiotherapists were filled out in June 2020 (ie, at the time of the lockdown of the French population in the context of the COVID-19 pandemic). The need for telecare was increased during and after the lockdown to prevent the spread of the virus while continuing to provide the necessary care to people with chronic diseases. In this context, an application decree was issued in April 2020, published in the Journal Officiel de la République Française on April 23, 2020, concerning telecare and aimed at facilitating its use by codifying its implementation and pricing [21].

Given this unprecedented health situation, technological devices such as the BYM tool, associated with a monitoring organization via telerehabilitation, have their place in an innovative reflection on the care pathways of patients with chronic diseases. Such digitally guided self-rehabilitation devices could enable real-time monitoring of self-rehabilitation by maintaining a link between the hospital and private sector and the patients’ homes. Long-term physical activity has now well-proven its effectiveness in slowing down the loss of autonomy in patients with neurodegenerative diseases [30,31]. Tools are to be developed to enable a slowing of this loss of autonomy by promoting motivation, which is often lacking in patients with Parkinson disease in connection with the impairment of dopaminergic systems. These tools can supplement the therapeutic rehabilitation arsenal, complementing other rehabilitation techniques without replacing them.

It should be noted that of all the independent physiotherapists contacted, few of them responded to the proposal to participate in this study. This shows the low enthusiasm of the private physiotherapists for the use of this tool and therefore a reluctance to change their practice. It has been shown that multidisciplinary management of patients with Parkinson disease was beneficial, with an overall improvement of motor skills, improved quality of life, and functional independence for daily life activities [32]. It is therefore important to be able to anchor this multidisciplinary practice in the health territory network via technological devices associated with telecare. During the study, many independent physiotherapists did not take advantage of this telemedicine offer, but their attitudes may have changed in the last few months [33].

### Conclusion

In conclusion, the acceptability, acceptance, and appropriation of the BYM tool are very high among private physiotherapists as well as patients, despite the lack of evolution of the tool during the study. It is important to present BYM as a rehabilitation and movement training tool and not as a substitute for physiotherapy management. This implies a global reflection on the place of new technologies within the patient’s care pathway, a real-time link between the hospital sector, the private sector, and the home. The recent health context requires a profound change in the organization of the care pathway for people with chronic diseases in particular, and this type of technological tool could accompany the increased use of telemedicine.

## Abbreviations

BYM: Beyond Your Motion
UPDRS: Unified Parkinson’s Disease Rating Scale
UPDRS M-EDL: Unified Parkinson’s Disease Rating Scale Motor Aspects of Experiences of Daily Living
UTAUT: Unified Theory of Acceptance and Use of Technology
